# On the design of a MEMS piezoelectric accelerometer coupled to the middle ear as an implantable sensor for hearing devices

**DOI:** 10.1038/s41598-018-22219-7

**Published:** 2018-03-02

**Authors:** A. L. Gesing, F. D. P. Alves, S. Paul, J. A. Cordioli

**Affiliations:** 1 Federal University of Santa Catarina, Mechanical Engineering, Florianopolis, 88040-900 Brazil; 2 Naval Postgraduate School, Monterey, CA 93943 USA

## Abstract

The presence of external elements is a major limitation of current hearing aids and cochlear implants, as they lead to discomfort and inconvenience. Totally implantable hearing devices have been proposed as a solution to mitigate these constraints, which has led to challenges in designing implantable sensors. This work presents a feasibility analysis of a MEMS piezoelectric accelerometer coupled to the ossicular chain as an alternative sensor. The main requirements of the sensor include small size, low internal noise, low power consumption, and large bandwidth. Different designs of MEMS piezoelectric accelerometers were modeled using Finite Element (FE) method, as well as optimized for high net charge sensitivity. The best design, a 2 × 2 mm^2^ annular configuration with a 500 nm thick Aluminum Nitride (AlN) layer was selected for fabrication. The prototype was characterized, and its charge sensitivity and spectral acceleration noise were found to be with good agreement to the FE model predictions. Weak coupling between a middle ear FE model and the prototype was considered, resulting in equivalent input noise (EIN) lower than 60 dB sound pressure level between 600 Hz and 10 kHz. These results are an encouraging proof of concept for the development of MEMS piezoelectric accelerometers as implantable sensors for hearing devices.

## Introduction

Over 10% of the world’s population is affected by hearing losses^1^, a disability which incurs in lower quality life and even reduced income. In most cases, the use of traditional hearing aids (HA) can help mitigate this disability. However, in patients whose inner ears are severely damaged, this approach may not be sufficient, and cochlear implants (CI) are the main alternative. In both hearing devices, the sound is acquired by one or more microphones and analyzed in a digital signal processor, both located in an external element near to or in the external ear. In the case of HA’s, the processed signal is amplified according to user needs and transmitted to the external ear by a loudspeaker (also called receiver). In CI’s, the signal is transmitted to an implantable element by a radio frequency antenna, and forward to the inner ear as a train of electrical impulses by means of an array of electrodes implanted in the cochlea. In both hearing devices, the visibility of the external elements is among user’s common complains due to discomfort and aesthetic issues. Besides, users cannot use most CI’s and HA’s whether underwater, during intense physical activities or even asleep. In this sense, totally implantable hearing devices have been proposed as an alternative to mitigate such constraints^2,3^, and the design of implantable sensors remains one of the main challenges to be overcame.

The development of a sensor for implantable hearing devices is a complex task in view of the very strict requirements for such a sensor, which are mainly related to the type of signal that must be provided to the hearing device and its working conditions. Such requirements have been thoroughly discussed in the literature, and the critical ones are: (i) usable bandwidth from 250 Hz to 8 kHz; (ii) dynamic range from 40 dB to 100 dB for input sound pressure level (SPL) for a reference pressure of 20 *μ*Pa applied to the tympanic membrane^4^; (iii) reduced dimensions in the case of sensors implanted in the middle ear, with dimensions not exceeding 2 × 2 × 2 mm^3 ^^5^; and (iv) reduced energy consumption, ideally smaller than 1 mW^4^. Such tight constraints have driven research into two main categories of implantable sensors: (a) subcutaneous microphones and (b) sensors directly coupled to the ossicular chain of the middle ear. In Table 1, bandwidth, equivalent input noise (EIN), power consumption and dimensions of the main current implantable sensors are summarized.

**Table 1:** Summary of current implantable sensor designs and performance.

Sensor by	Technology	Bandwidth (kHz)	EIN SPL	Power	Dimensions
Carina®^6^	Subcutaneous Capacitive Microphone	0.2–5.0	30 dB	0.25 mW	—
Park *et al*.^8^	Piezoresistive MEMS Accelerometer	0.7–8.0	65 dB	>1 mW	0.3 mm × 0.4 mm
Ko *et al*.^4^	Capacitive MEMS Displacement Sensor	0.6–8.0	50 dB	4.5 mW	2.0 mm × 2.0 mm
Sachse *et al*.^5,29^	Capacitive MEMS Displacement Sensor	0.5–5.0	40 dB	—	2.0 mm × 2.0 mm
Zurcher *et al*.^9^	Capacitive MEMS Accelerometer	0.2–5.0	60 dB	4.5 mW	1.0 mm × 1.0 mm
Esteem^7^	Piezoelectric Force Transducer	0.2–8.0	—	—	—
Yip *et al*.^10^	Piezoelectric Force Transducer	0.3–5.2	60 dB	0.01 mW	—
Beker *et al*.^11,12^	Piezoelectric MEMS Accelerometer	0.5–2.5	—	—	4.2 mm × 4.0 mm
Jia *et al*.^13,14^	Floating Piezoelectric Microphone	0.5–8.0	50 dB	—	5.9 mm × 2.4 mm

Both categories are in use by commercial hearing devices with performance issues reported in literature. A subcutaneous microphone is used by the implantable hearing aid Carina^6^, while the Esteem device^7^ adopts a piezoelectric force transducer coupled to the middle ear. Subcutaneous microphones have been associated with large sensitivity variability (due to variations in skin thickness), dermatological problems and the need for specific signal processing techniques to minimize the influence of body noises^6^. On the other side, different concepts of middle ear implantable sensors have been proposed, but without successfully fulfilling of all requirements as briefly detailed below.

Park *et al*.^8^ developed a 287 *μ*m × 387 *μ*m × 230 *μ*m piezoresistive MEMS accelerometer, which performed well for frequencies between 700 Hz and 8 kHz only for stimulation above 65 dB SPL, and with power consumption over 1 mW. Ko *et al*.^4^ and Sachse^5^ proposed the use of MEMS capacitive displacement sensors. Ko’s transducer was able to detect sound signals in a frequency range from 600 Hz to 8 kHz above 50 dB SPL consuming over 4.5 mW, whereas Sachse’s sensor performed well between 500 Hz and 5 kHz for signals above 40 dB SPL. Power consumption, however, was not reported for Sachse’s sensor. Alternatively, Zurcher *et al*.^9^ developed a 1.0 mm × 1.0 mm Capacitive MEMS accelerometer, which was capable of measuring sounds between 200 Hz and 6 kHz for SPLs superior to 55 dB, however resulted in a heavy sensor (25 mg) which may affect the dynamic of the middle ear and large power consumption (4.5 mW).

The commercially available Esteem device^7^, and the authors Yip *et al*.^10^ and Beker *et al*.^11^ resorted on the piezoelectric effect as an approach to reduce power consumption, which is intrinsically higher in capacitive and piezoresistive sensors. Yip *et al*.^10^ used lead zirconate titanate (PZT) beams - a similar strategy to the one employed by in the Esteem device^7^ - to test a series of charge amplifiers and sound processing strategies concerning implantable hearing devices. Yip’s force transducer exhibits functional bandwidth from 300 Hz to 5.2 kHz for SPL superior to 60 dB SPL; the authors, however, do not provide details of the sensor design and suggest that it should be reduced to fit in the middle ear cavity. Beker *et al*.^11,12^ proposes a piezoelectric MEMS accelerometer as an implantable sensor for hearing devices. The reported sensor exhibits large dimensions (4.2 mm × 4.0 mm) and lacks experimental analysis. Jia *et al*.^13,14^ developed a PZT floating piezoelectric microphone (FPM) encapsulated in a titanium packaging. This sensor is 5.9 mm × 2.4 mm and was able to detect 50 dB SPL between 500 Hz and 8.0 kHz, although its dimensions must be further reduced for the sensor to be implantable in the middle ear cavity.

In this paper, a feasibility analysis of an Aluminum Nitride (AlN) based MEMS piezoelectric accelerometer as a sensor for implantable hearing devices is reported. Although AlN piezoelectric effect is weaker than PZT and other piezoelectric ceramics in layers of similar thicknesses, AlN is more suitable for MEMS fabrication processes, and usually results in higher quality factors and reduced thermal noise^15^. Thus, it is expected that the development of an AlN based piezoelectric accelerometer ought to combine high quality factors and low thermal noise, low power consumption, large bandwidth and superior dynamic range. To perform this analysis, Finite Element (FE) models of the MEMS piezoelectric accelerometers were developed, along with a procedure to estimate the sensor noise floor. Optimization techniques were then applied to maximize the sensors’ sensitivities within the frequency range of interest, and the best design was manufactured and characterized. The results were used to validate the numerical model of the sensor, which was later coupled to a FE model of the human middle ear with the aim of providing the necessary information for the feasibility analysis, in view of the current state of the art shown in Table 1.

## Modeling of the MEMS piezoelectric accelerometer

### Finite Element model

**Figure 1:**
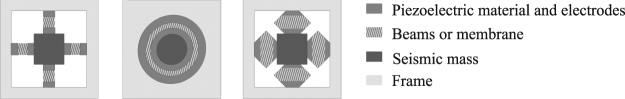
Top view of the different MEMS piezoelectric accelerometers analyzed: trampoline (left), annular (middle) and hexagonal beams with square seismic mass (right).

Several designs of MEMS piezoelectric accelerometers can be found in the literature^15–19^, however, except for Beker *et al*.^11^, different applications than implantable sensors have been considered. In general, these sensors are comprised of five elements: frame, seismic mass, a set of beams to provide the system stiffness, a piezoelectric layer and electrodes. For the present study, three configurations have been chosen in view of the sensitivity observed in previous works and include: (i) a traditional trampoline geometry, (ii) an annular geometry, and (iii) an alternative design composed of four hexagonal beams and a square seismic mass^19^, as shown in Fig. 1. A common characteristic among these configurations is that two piezoelectric regions are included, one located closer to the frame (outer layer) and the other located close to the seismic mass (inner layer). While one region is under tensile stress, the other is under compressive stress, so that charge difference from both layers enhances the net charge *Q*_T_ generated by the piezoelectric sensor.

**Figure 2:**
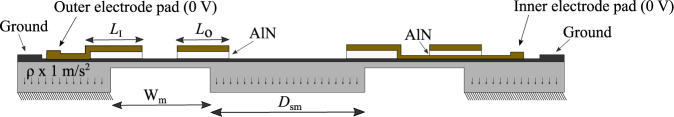
Geometric parameters, boundary conditions and body force imposed in the FE model, here represented in a section view of the annular accelerometer.

Due to the complex nature of MEMS piezoelectric accelerometers, FE modeling has been the preferred method for simulation of these devices, having proved to generate reliable results in numerous studies^15,16,18^. Since piezoelectricity originates from the coupling between mechanical and electrical behaviors of the structure, both boundary conditions ought to be set in the FE model. Electrically, piezoelectric accelerometers may be designed to operate as electrical charge or voltage sources. The charge source mode provides a significant advantage over the voltage source, since parasitic and cable capacitance do not interfere with the net charge response of the sensor. Thus, the amplifier circuit can be placed distant from the sensor without signal losses^20^, which may be crucial for middle ear implantable sensors. Hence, the charge source mode was selected. In the FE model, this mode is obtained by forcing a zero voltage difference between the ground and top planar electrodes^15^. The net charge response *Q*_T_ of the accelerometer is then calculated by^20^

1 $${Q}_{{\rm{T}}}={\int }_{{A}_{{\rm{I}}}}D\,{\rm{d}}{A}_{{\rm{I}}}-{\int }_{{A}_{{\rm{O}}}}D\,{\rm{d}}{A}_{{\rm{O}}},$$

where *D* represents the electric displacement, and *A*_I_ and *A*_O_ are the inner and outer electrode areas (see Fig. 2).

Mechanically, the sensor is considered crimped at the base of the frame, and a harmonic force *ρ* × 1 m/s^2^ is applied to the entire domain to emulate an unitary acceleration. Figure 2 shows the section view of an annular piezoelectric accelerometer where the boundary conditions and body forces applied in the Fe-models are represented. The geometric parameters of the annular accelerometer are also shown in the same figure, where: *D*_sm_ stands for the seismic mass diameter, *W*_m_ is the membrane width, *L*_O_ is the outer AlN layer length and *L*_I_ stands for the inner AlN layer length. Further details on the mesh and material properties of the FE models are presented on the methods section.

The FE models were developed considering design rules imposed by MEMSCAP’s Piezoelectric multi-users MEMS Processes (PiezoMUMPS). The PiezoMUMPS procedure starts with a Silicon on Insulator (SOI) wafer; which consists of a stack of 400 *μ*m thick handle wafers, 1 *μ*m thin buried oxide and a 10 *μ*m thick SOI device layer. A 500 nm AlN layer is sputtered and patterned on top of the SOI, upon which two metal layers - 1 *μ*m thick Aluminum and 0.02 *μ*m thin Chromium - are deposited. The final steps of the process are the removal of the silicon on the top of the SOI layer via reactive ion etching (RIE) and removal of the wafer’s bottom silicon layer via deep reactive ion etching (DRIE)^21^. Both foundry and process were selected due to the lower cost, fast prototyping and reliability. However, it is important to remark that the 500 nm AlN layer is a strong restriction to the piezoelectric accelerometer’s performance, which, for the sake of this proof of concept, was considered acceptable. Taking in account a maximum area of 2 × 2 mm^2^ and the PiezoMUMPS restrictions, the minimum, maximum and fixed values of the parameters of the annular accelerometer defined for the analysis are shown in Table 2.

**Table 2:** Minimum, maximum and fixed parameters applied to the annular accelerometer model due to the design rules and the middle ear ossicular chain dimensions.

Fixed parameter	Value	Variable parameter	Minimum value	Maximum value
Seismic mass and frame thickness	411 *μ*m	*D*_sm_	100 *μ*m	1500 *μ*m
Membrane thickness	10 *μ*m	*W*_m_	200 *μ*m	900 *μ*m
AlN thickness	0.5 *μ*m	*L*_O_	25 *μ*m	850 *μ*m
Electrode thickness	1.02 *μ*m	*L*_I_	25 *μ*m	850 *μ*m

The values provided in Table 2 also define the inferior and superior bounds applied in the optimization process. Besides these limits, the optimization process also considered two linear constraints given by

2 $${D}_{{\rm{sm}}}+2{W}_{{\rm{m}}}\le 1.8\,{\rm{mm}}\,{\rm{and}}\,{W}_{{\rm{m}}}-{L}_{{\rm{O}}}-{L}_{{\rm{I}}}\ge 25\,\mu {\rm{m}}.$$

These constraints imposed a maximum 2 × 2 mm^2^ sensor while keeping 100 *μ*m on each side for trails and bonding pads and forced a gap of at least 25 *μ*m between the two AlN layers, which avoids short circuiting. In the case of the trampoline and hexagonal beams designs, these constraints and limits were slightly altered to reflect these sensor’s characteristics.

### Sensor internal noise

A key comparison factor for hearing devices sensors installed in the middle ear is the transducer’s equivalent input noise (EIN) in SPL, i. e the minimum detectable external acoustic field (what the sensor is actually aiming to detect) due to the sensor internal noise. This lower limit of the dynamic range for implantable sensors has been defined previously as 40 dB SPL at the tympanic membrane for hearing devices^5^. At this level of excitation, the acceleration at the middle ear ossicular chain reaches 1 mm/s^2^ at 1 kHz at the most sensitive position. Hence, it is necessary to take special attention to the modeling of this AlN sensor’s internal (inherent) noise. In the case of a piezoelectric accelerometer, the internal noise ${\overline{\ddot{Y}}}_{{\rm{N}}}$ in terms of equivalent acceleration spectra can be estimated as the sum of three components as^22^

3 $${\overline{\ddot{Y}}}_{{\rm{N}}}^{2}={\overline{\ddot{Y}}}_{{\rm{Th}}}^{2}+{\overline{\ddot{Y}}}_{{\rm{El}}}^{2}+{\overline{\ddot{Y}}}_{{\rm{Amp}}}^{2},$$

where ${\overline{\ddot{Y}}}_{{\rm{Th}}}$ is the spectral acceleration due to thermal noise, ${\overline{\ddot{Y}}}_{{\rm{El}}}$ is the spectral acceleration due to electrical noise and ${\overline{\ddot{Y}}}_{{\rm{Amp}}}$ is the acceleration spectral noise due to the amplifier circuit. The thermal source ${\overline{\ddot{Y}}}_{{\rm{Th}}}$ behaves similar to a white noise up to 80 GHz^15^ and is given by^23^

4 $${\overline{\ddot{Y}}}_{{\rm{Th}}}^{2}=4\,{\kappa }_{{\rm{B}}}\,T\,\frac{2\pi {f}_{{\rm{n}}}}{{m}_{{\rm{ms}}}\,Q},$$

where *κ*_B_ is the Boltzmann constant (1.3807 ⋅ 10^−23^ J/K), *T* is the temperature in Kelvin, *f*_n_ is the natural frequency, *m*_ms_ is the seismic mass and *Q* is the quality factor. In general, ${\overline{\ddot{Y}}}_{{\rm{Th}}}$ is higher at MEMS sensors than in macro accelerometers due to its smaller seismic mass. AlN sensors, however, usually exhibit higher quality factors than PZT accelerometers, therefore rendering lower ${\overline{\ddot{Y}}}_{{\rm{Th}}}$ for similar *f*_n_ and *m*_sm_.

Electrical noise occurs as random energy variation due to the capacitance of each electrode set, which generates a flicker noise behavior^15^. Its spectral acceleration ${\overline{\ddot{Y}}}_{{\rm{El}}}$ in a piezoelectric sensor with two electrodes is the sum of the contributions of the outer and inner capacitances ${\overline{\ddot{Y}}}_{{{\rm{El}}}_{{\rm{Outer}}}}$ and ${\overline{\ddot{Y}}}_{{{\rm{El}}}_{{\rm{Inner}}}}$, or^23^,

5 $${\overline{\ddot{Y}}}_{{\rm{El}}}^{2}={\overline{\ddot{Y}}}_{{{\rm{El}}}_{{\rm{Outer}}}}^{2}+{\overline{\ddot{Y}}}_{{{\rm{El}}}_{{\rm{Inner}}}}^{2}=4\,{\kappa }_{{\rm{B}}}\,T\,\frac{\eta {C}_{{\rm{O}}}}{2\pi f{Q}_{{\rm{O}}}{(f)}^{2}}+4\,{\kappa }_{{\rm{B}}}\,T\,\frac{\eta {C}_{{\rm{I}}}}{2\pi f{Q}_{{\rm{I}}}{(f)}^{2}},$$

where *η* is the capacitive loss factor, *C*_I_ and *C*_O_ are the capacitance of inner and outer electrodes, and *Q*_O_(*f*) and *Q*_I_(*f*) are the charge per acceleration frequency response obtained on each electrode set. It is important to note that the original formulation by Levinzon^22,23^ does not account for the frequency behavior of the charge response, which was included here.

Lastly, the spectral acceleration noise caused by the amplifier circuit ${\overline{\ddot{Y}}}_{{\rm{Amp}}}$ on sensors with two electrodes sets may be calculated as^22^

6 $${\overline{\ddot{Y}}}_{{\rm{Amp}}}^{2}={\overline{\ddot{Y}}}_{{{\rm{Amp}}}_{{\rm{Outer}}}}^{2}+{\overline{\ddot{Y}}}_{{{\rm{Amp}}}_{{\rm{Inner}}}}^{2}={[\frac{{e}_{{\rm{O}}}(f){C}_{{\rm{O}}}}{{Q}_{{\rm{O}}}(f)}]}^{2}+{[\frac{{e}_{{\rm{I}}}(f){C}_{{\rm{I}}}}{{Q}_{{\rm{I}}}(f)}]}^{2},$$

where ${\overline{\ddot{Y}}}_{{{\rm{Amp}}}_{{\rm{Outer}}}}$ and ${\overline{\ddot{Y}}}_{{{\rm{Amp}}}_{{\rm{Inner}}}}$ are the spectral acceleration noise due to the amplifier circuit applied to each electrode set, and *e*_O_(*f*) and *e*_I_(*f*) are the voltage spectral noise of the electronic circuits applied to the sensor by the circuit noise at each electrode. In this study the amplifier noise was neglected.

## Results

### Optimization results

An optimization process was applied with the goal of maximizing the minimum net charge *Q*_T_ in the frequency range between 250 Hz and 8 kHz. This process was performed using the genetic algorithm (GA), available in Mathwork’s Matlab programming language^24,25^. In GA, natural selection is emulated, i. e. an elite is selected from a population based on a fitness ranking, and reproduced through procedures such as crossover and mutation. GA has proven to be a reliable optimization tool which can deal with large number of variables and complex cost surfaces^26^. In the annular and trampoline model optimization, initial population was composed of 50 individuals, while for the hexagonal beams sensor initial populations was set to 200^25^. For all cases, crossover fraction was 0.8, elite was 5% of the population, and optimization converged when a difference of 10^−6^ was achieved between 50 stall generations, or terminated when the number of iterations reached 500 generations. Table 3 shows the results for the three optimized accelerometers. It can be seen that, although the annular sensor exhibits the largest charge response among the three sensors, its spectral noise at 1 kHz is also the largest, due to its higher capacitance. The hexagonal beams sensor, on the other hand, has lower spectral noise at 1 kHz, which occurs due to its lower capacitances, whereas its charge sensitivity is only slightly higher than the trampoline’s. In the three sensors, resonance frequencies *f*_n_ are larger than the superior limit of the frequency range (8 kHz), which occurs due to the small size and high stiffness of these designs.

**Table 3:** Parameters of the three accelerometers types obtained through the optimization process for maximum charge sensitivity in the frequency range. *Q*_I_, *Q*_O_ and ${\overline{\ddot{Y}}}_{{\rm{N}}}$ are evaluated at 1 kHz.

Sensor	*f*_n_	*m*_sm_	*Q*_O_	*Q*_I_	*C*_O_	*C*I	${\overline{\ddot{Y}}}_{{\rm{N}}}$
Trampoline	18.0 kHz	0.61 mg	2.5 fC s^2^/m	2.6 fC s^2^/m	51 pF	59 pF	6.6 mm/s^2^/(Hz)^1/2^
Annular	18.9 kHz	0.53 mg	3.8 fC s^2^/m	3.9 fC s^2^/m	194 pF	138 pF	7.7 mm/s^2^/(Hz)^1/2^
Hexagonal beams	16.8 kHz	1.00 mg	2.7 fC s^2^/m	2.7 fC s^2^/m	33 pF	44 pF	5.3 mm/s^2^/(Hz)^1/2^

Due to the higher net charge exhibited by the annular accelerometer, it was opted to select this sensor for prototyping and experimental analysis, despite its higher inherent noise. This choice increases the probability of successful experimental measurements of the charge behavior and even of the spectral noise, which are crucial to prove the concept. The selected dimensions of annular accelerometer prototype’s are: *D*_sm_ = 840 *μ*m, *W*_m_ = 480 *μ*m, *L*_I_ = 210 *μ*m and *L*_O_ = 235 *μ*m (see Fig. 2).

**Figure 3:**
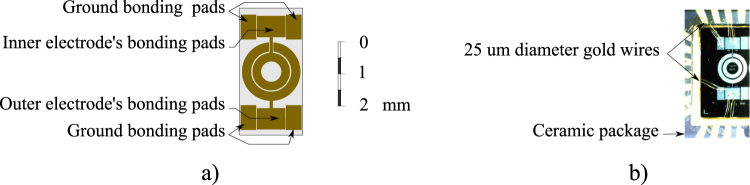
(**a**) Top view of the annular accelerometer (prototype dimensions). (**b**) Accelerometer prototype fixed to the ceramic packaging and electrically connected via 25 *μ*m diameter gold wires.

The manufacture process involved the definition of five mask layouts for the selected sensor, which are shown in the Methods section, and were later forward to MEMSCAP for production. A representation of the top view of the annular accelerometer is shown in Fig. 3a, where the ground, inner and outer electrodes’ bonding pads are highlighted. The bonding pads were made larger than the minimum necessary to facilitate wire bonding and mounting of the sensor. Therefore, the overall die sensor ended up occupying 2 mm × 4 mm instead of the original 2 mm × 2 mm. Prototypes were fixed to ceramic packages provided by NTK technologies, and electrical connections were made through 25 *μ*m diameter gold wires, as shown in Fig. 3b.

### Experimental results

**Figure 4:**
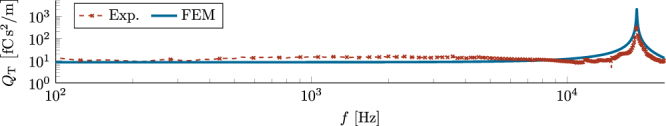
Simulated and experimentally measured net charge *Q*_T_ of the optimized annular sensor.

Experiments were performed to characterize the sensor’s net charge response and spectral noise frequency behavior. Details of the experimental setup are reported in the Methods section. Figure 4 shows the net charge *Q*_T_ of the prototype obtained experimentally and through the FE model for a frequency range of 100 Hz to 24 kHz, where a very good agreement can be observed. A minor difference between the projected 18.9 kHz resonance frequency to the experimental 19.1 kHz may be noted, which happened most likely due to small variations in the commercial fabrication process.

**Figure 5:**
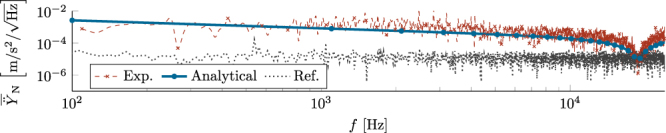
Experimental and analytical spectral acceleration noise of the prototype and of the reference accelerometer.

The sensor spectral acceleration ${\overline{\ddot{Y}}}_{{\rm{N}}}$ was measured using a ultra low noise charge amplifier (details in the methods section), and it is compared to the analytical estimative in Fig. 5. This estimative was made considering: (i) the experimentally obtained charge presented in Fig. 4, (ii) measured capacitances of *C*_O_ = 340 pF and *C*_I_ = 270 pF, which differ from FE values (Table 3) due to parasitic capacitances of the trails and bonding pads, (iii) a quality factor *Q* = 100 obtained by means of the half-power bandwidth method applied to the experimental data showed in Fig. 4, and (iv) an estimated capacitive loss factor *η* = 0.01. Figure 5 shows the sensor spectral acceleration noise obtained experimentally and analytically, together with the measured spectral acceleration noise of the reference accelerometer, which guarantees that the prototype’s measured noise is its inherent noise, and not environment influences. The accuracy of the analytical prediction is observable, especially the noise decrease near the natural frequency *f*_n_ of the sensor, whereas the original formulation by Levinzon^23^ neglects this behavior.

### Sensor performance

**Figure 6:**
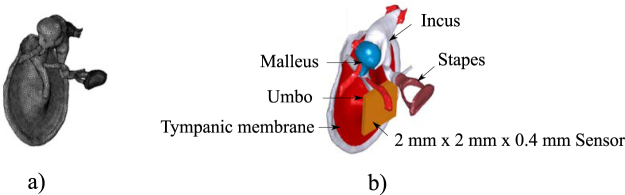
(**a**) Mesh used in the FE model of the human middle ear. (**b**) Representation of a 2 × 2 × 0.4 mm^3^ MEMS sensor positioned at the Umbo.

In order to estimate the sensor’s EIN when implanted in the ossicular chain, a middle ear FE model developed by Pires *et al*.^27^ was considered. Figure 6a shows the mesh used in the FE model. The middle ear model allows to estimate the normal direction acceleration of any point of the middle ear ossicular chain for a given sound pressure applied at the tympanic membrane. This information can then be used to predict the sensor EIN relative to the acoustic field incident at the tympanic membrane when the sensor is installed at different positions. The Umbo (part of the Malleus connected to the tympanic membrane, see Fig. 6b) has been the preferred installation site in previous works which developed sensors of similar dimensions^4,9,11,13^, since the acceleration levels are higher than at any other point of the ossicular chain. Furthermore, it is the location which enables the application of larger sensors (up to 2 × 2 mm^2^), which makes it ideal for prototype testing. Surgically, however, this location is not considered appropriate due to its difficult access^28^, which may lead to higher risk of facial nerve damage during the surgical procedure. This is the reason why other positions at the ossicular chain are usually considered for smaller sensors. For comparison purposes, it was opted to use the Umbo as the sensor site in this analysis.

**Figure 7:**
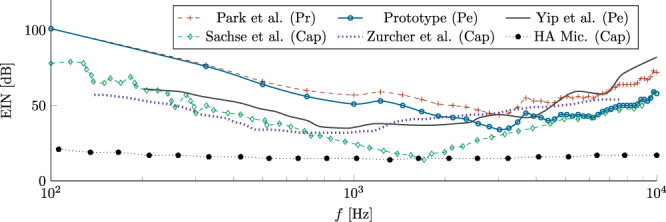
EIN of the piezoelectric (Pe) prototype, and other capacitive (Cap) and piezoresistive (Pr) implantable sensors and a capacitive HA Microphone. In all implantable sensors 100 Hz frequency discretization is applied, while third octave band is used for the HA microphone.

In this study, EIN was estimated based on the experimental acceleration noise spectrum shown in Fig. 5 and the middle ear FE model. A frequency discretization of 100 Hz was applied to the spectral noise ${\overline{\ddot{Y}}}_{{\rm{N}}}$, which is a common discretization used for CIs (most of them acquire and process signal for periods of 10 ms). The FE model was used to estimate the corresponding acoustic pressure at the tympanic membrane that would result in the sensor spectral noise (in terms of acceleration) at the Umbo. This can be done by inverting the the frequency response function in terms of acceleration at the Umbo due to an acoustic pressure at the tympanic membrane and multiply by the sensor experimental acceleration noise spectrum. The result of this procedure for the piezoelectric (Pe) annular prototype is shown in Fig. 7, alongside the EIN of other implantable sensors.

In the case of the Capacitive (Cap) MEMS displacement sensor of Sachse *et al*.^5,29^ and the Capacitive MEMS accelerometer of Zurcher *et al*.^9^, the authors measured its EIN experimentally. Coupling the sensor to the Umbo of a temporal bone, both authors would, then, actuate on the tympanic membrane with a harmonic sound wave. Signal was acquired applying certain spectral discretization, which Sachse opted for a 100 Hz discretization, and Zurcher opted for 200 Hz. For this comparison we opted for converting zurcher’s EIN to 100 Hz discretization. Regarding Yip’s piezoelectric force transducer^10^ and Park’s MEMS piezoresistive (Pr) accelerometer, only their spectral acceleration noise were available, since the authors did not directly analyze their sensors’ EIN. Therefore, the same approach used for the current prototype was adopted to estimate the EIN. For comparison purposes, the EIN of a hearing aid electret condenser microphone (ECM) is also shown in Fig. 7. Differently than CIs, hearing aids usually consider third-octave bands discretization in noise analysis^30^, which was maintained.

The current AlN sensor prototype exhibits a better performance when compared to the piezoresistive MEMS accelerometer developed by Park^8^, showing a broader bandwidth for lower EIN. It is important to note that the sensors compared in Fig. 7 do not have the same size. In fact, they are mostly larger than the current prototype, except for Park’s, which reports on an ultra-miniature implantable sensor (0.3 mm × 0.4 mm), and Zurcher’s MEMS capacitive accelerometer (1.0 mm × 1.0 mm). Compared to the capacitive transducers, the MEMS AlN accelerometer prototype shows an inferior performance at frequencies below 3 kHz, but for higher frequencies the performance is similar to the most sensitive sensor, which is Sachse’s MEMS capacitive displacement sensor (2.0 mm × 2.0 mm). Further on, it can be seen that the AlN prototype sensor is outperformed by other piezoelectric sensors. While Jia’s microphone^13^ measures down to 50 dB SPL from 500 Hz to 8 kHz, and Yip’s^10^ force transducer is able to detect 60 dB SPL with bandwidth from 300 Hz to 5.2 kHz, the current MEMS accelerometer can detect SPL only above 60 dB between 600 Hz and 10 kHz. One of the reasons for that is the size; Jia’s sensor measures 5.9 mm × 2.4 mm, while Yip reports that his sensor should be further reduced to fit in the middle ear cavity. The current sensor, on the other hand, although still large, is among the smallest developed so far. At last, it is evident that no implantable sensor has yet came close to the performance of HA’s electret condenser microphone. This, however, is expected, since implantable sensors are yet on the early stages of development, while electret microphones have been under development for decades.

## Discussion

In this investigation, a thin 500 nm layer of AlN was chosen as piezoelectric material due to the availability of commercial fabrication, fast prototyping and higher quality factors^15^. A design methodology was established using FE simulation as well as optimization by means of a genetic algorithm. Figure 4 shows that the FE model developed can very accurately predict the net charge response of the piezoelectric accelerometer. Regarding capacitive prediction, however, there was a small variation, due most likely to parasitic capacitances in the trails and bonding pads of the prototype. Characterization and analysis were combined with the prediction of the sensor’s EIN performance when implanted in the middle ear in order to allow comparison and insights for future designs. Figure 7 shows that, although design rules and fabrication constraints imposed by the foundry forced severe restriction on the design that impacted the overall performance, preliminary results reported in this article are encouraging and suffice to validate the design method and proof the concept.

Furthermore, it is clear that noise reduction strategies should be applied to increase the MEMS piezoelectric accelerometer’s performance. For instance, Table 3 shows that, although the annular accelerometer exhibited higher net charge, its high capacitance drastically increased its spectral acceleration noise. Besides, Fig. 7 shows that, the main drawback of our AlN device is its poor performance at frequencies up to 2 kHz. Hence, next generation sensor’s should be designed considering the low acceleration exhibited by the middle ear ossicular chain at this frequency range. In addition, the frequency behavior of the spectral noise acceleration ${\overline{\ddot{Y}}}_{{\rm{N}}}$ (see Fig. 5) should be used as a tool for noise reduction at low frequencies, similarly to the strategy applied by Sachse^5,29^ with his capacitive MEMS displacement sensor.

Moreover, one of the most restrictive elements of this sensor was the necessary use of the 500 nm AlN piezoelectric layer. Typically, AlN exhibits high quality factor and low charge sensitivity, which decreases the thermal noise ${\overline{\ddot{Y}}}_{{\rm{Th}}}$ while increasing the electrical noise ${\overline{\ddot{Y}}}_{{\rm{El}}}$. From Fig. 5, it is clear by the flicker behavior of ${\overline{\ddot{Y}}}_{{\rm{N}}}$ that the electrical noise is the main noise source on this sensor in this frequency range. Actually, ${\overline{\ddot{Y}}}_{{\rm{Th}}}$ is around 10^−6^ m/s^2^/(Hz)^1/2^ over the entire frequency range, and the corner frequency (on which these two noise sources are equal) is above 24 kHz. Therefore, the use of AlN for small noise at this frequency range may not be justified. Other piezoelectric material, such as PZT, which yields lower quality factors and higher piezoelectric coefficients, should render lower electrical noise, therefore lower noise floor and EIN, allowing further size reduction and making this approach very attractive.

Further challenges remain to make piezoelectric MEMS accelerometers ready for implantation in the middle ear, such as low noise charge to voltage amplification, bio-compatibility and low form factor packaging. Experiments in temporal bones should be performed, and finally *in vivo* tests ought to be successful and prove the functionality of the device in the long term.

## Methods

### FE model

**Figure 8:**
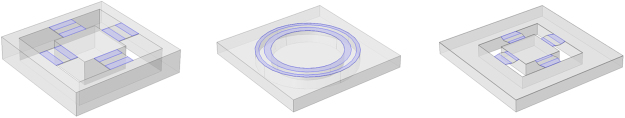
3D layouts of the three MEMS piezoelectric accelerometers considered modeled in Comsol Multiphysics: trampoline on the left, annular model at the center and the hexagonal beams with a square seismic mass accelerometer on the right. The frame, beam and seismic mass are shown in light gray, whereas the AlN layers and electrodes are shown in blue.

Models of the three accelerometer designs were developed using the FEM software Comsol Multiphysics. Figure 8 shows the 3D layouts of the trampoline design on the left, annular on the center and hexagonal beams on the right. On the FE models the 1 *μ*m thick buried silicon oxide layer was neglected, being considered as silicon instead. The 0.2 *μ*m silicon oxide layer which was grown at the top of the wafer was also neglected. Viscous damping factor was assumed for all domains as 0.005.

Electrical and mechanical boundary conditions and a harmonic body force were applied in the FE model as shown in Fig. 2. Electrically, bottom and top surfaces of both AlN layers are considered grounded (0 V), which imposes the charge source behavior to the sensor. Mechanically, the bottom surface of the frame is considered crimped, and the whole body is subject to an harmonic force equals *ρ* × 1 m/s^2^. The material properties were all obtained from the Comsol Multiphysics material library, which is very accurate for piezoelectric materials. For AlN this means that the permittivity matrix is

7 $$\varepsilon =[\begin{array}{ccc}9 & 0 & 0\\ 0 & 9 & 0\\ 0 & 0 & 9\end{array}]\times \mathrm{8,854}\times {10}^{-12}\,\frac{{\rm{F}}}{{\rm{m}}},$$

the coupling matrix is

8 $${\bf{e}}=[\begin{array}{llllll}0 & 0 & 0 & 0 & -0.4 & 0\\ 0 & 0 & 0 & -0.4 & 0 & 0\\ -0.58 & -0.58 & 1.55 & 0 & 0 & 0\end{array}]\,\frac{{\rm{C}}}{{{\rm{m}}}^{2}},$$

and the elasticity matrix is

9 $${\bf{c}}=[\begin{array}{llllll}410 & 149 & 99 & 0 & 0 & 0\\ 149 & 410 & 149 & 0 & 0 & 0\\ 99 & 149 & 410 & 0 & 0 & 0\\ 0 & 0 & 0 & 125 & 0 & 0\\ 0 & 0 & 0 & 0 & 125 & 0\\ 0 & 0 & 0 & 0 & 0 & 125\end{array}]\,{\rm{GPa}}.$$

For Silicon relative permittivity *ε*_R_ was 4.5, density *ρ* = 2320 kg/m^3^, Young’s Modulus *E* = 160 GPa and Poisson’s ratio *ν* was 0.22. For Aluminum, *ε*_R_ = 1, *ρ* = 2700 kg/m^3^, *E* = 70 GPa and *ν* = 0.33. During optimization, all simulations were performed from 250 Hz to 8 kHz with a 50 Hz discretization, while for experimental comparisons (Fig. 4) frequency range was from 100 Hz to 24 kHz with 20 Hz discretization.

### Fabrication

**Figure 9:**
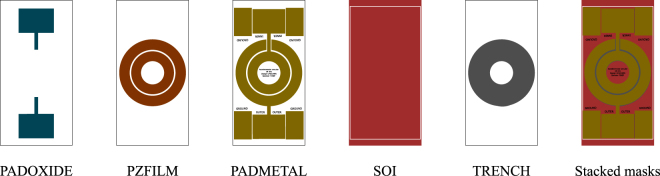
Sequence of five masks sent to MEMSCAP for fabrication, and on the right the five masks are shown stacked.

Figure 9 shows the five masks created using the software MEMSPro and sent to MEMSCAP for fabrication. The silicon dioxide layer is patterned via the PADOXIDE mask, which is designed to provides electrical isolation for the electrodes, trails and bonding pads, while maintaining the bottom surfaces of both AlN layers clear for mutual grounding. The 500 nm thick AlN layer is sputtered using the PZFILM mask, where a gap in the outer layer of AlN is made so that the inner electrode’s trail may pass. This gap is made large enough to prevent short-circuiting between the two electrodes. The electrode is patterned through the PADMETAl mask, which, besides developing the inner and outer electrodes, also patterns the ground bonding pads and marks the sensor’s characteristics. The fourth mask in the fabrication process is the SOI, with which the top layer of 10 *μ*m silicon is patterned. In the prototype, only the sensor’s dimensions of 2 mm × 2 mm was delimited with this mask. Finally, with the TRENCH mask the seismic mass is developed in the DRIE process in the back of the wafer, which removes 400 *μ* thickness of the SOI at the marked ring. On the right in Fig. 9 all five stacked masks are shown. Both AlN and silicon oxide layer are hidden, since they are entirely covered by the electrodes, defined by the PADMETAL mask.

### Experimental setup

Prototypes arrived from MEMSCAP attached to a thermal tape, which released the MEMS sensors without damaging them when heated to 150 °C. Prototypes were then fixed to a ceramic packaging provided by NTK technologies. The ceramic packages assured the stiffness of the mounting setup, which guaranteed the smooth response shown in Fig. 4. In previous setups, a more flexible mounting had been tested and results were unsatisfactory, since mounting resonance frequencies could be identified in the net charge response and coherence was low. Further on, ceramic packages were perforated in its center using diamond drill bits, to provided a back cavity that allowed the seismic mass to move freely. In addition, these ceramic packages have bonding pads which are compatible to standard gold wire bonding processes. Wires were soldered to the outer pads of the ceramic package to allow connections with the born-BNC adapters fixed on the experimental structure. Later on, the prototype were attached to the packages, and the wire bonding operation was then made via wedge bonding using the TPT HB02 wire bonder. This operation was performed at 120 °C.

**Figure 10:**
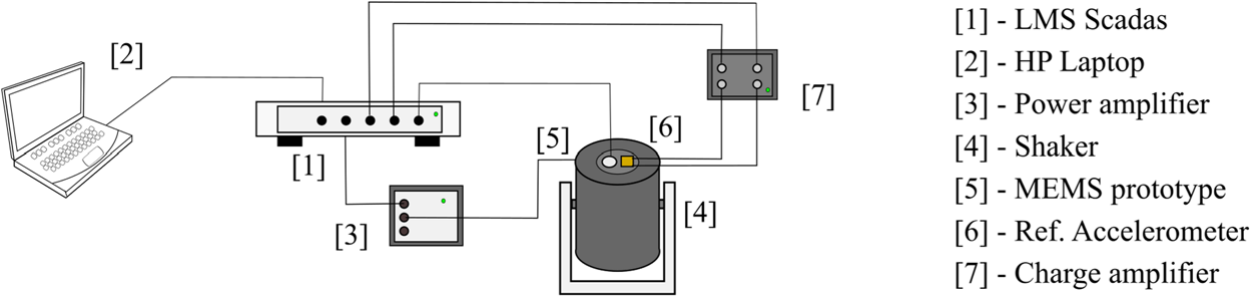
Experimental setup for the net charge measurement of our MEMS AlN accelerometer.

Figure 10 shows the experimental setup used to measure the net charge response *Q*_T_ generated by the MEMS prototype. A LMS Siemens Scadas SCM-V8-E signal analyzer^1^, which was controlled via a notebook^2^, generated a white noise from 2 Hz to 32 kHz. This white noise was then sent to a B&K 2716- C power amplifier^3^, which besides amplifying voltage, acted as a current limiter for the a B&K 4810 electrodynamic shaker^4^. The recommended frequency range of operation for the shaker is up to 20 kHz. To minimize structural vibration transmission into the measurement setup, the shaker was suspended by steel cables fixed to a steel structure. On the shaker table, a B&K 4519 reference accelerometer^5^ and the MEMS prototype^6^ were attached as close as possible to each other, which ensured the same acceleration was being applied to both sensors. Voltage response of the reference accelerometer was acquired by the signal analyzer, while the MEMS prototype’s charge response would go to an ultra-low noise B&K Nexus 2692-C charge amplifier^7^, which is able of measuring sensors sensitivities as low as 0.1 aC/m/s^2^ (10^−19^ C/m/s^2^). These procedures resulted in a coherence near one up to 20 kHz, where it started to decrease due to the shaker limitation. Data was acquired from 2 Hz to 32 kHz, with a 1 Hz discretization, and average were calculated considering 40 samples for each measurement. In these analysis, charge of the inner *Q*_I_ and of the outer *Q*_O_ AlN layers were acquired simultaneously, and the net charge *Q*_T_ was calculated based on these measurements. To measure the spectral noise density ${\overline{\ddot{Y}}}_{{\rm{N}}}$ of the AlN MEMS accelerometer, a procedure similar to the net charge measurement (Fig. 10) was developed. However, in this experiment the power amplifier^2^ and the shaker^3^ were replaced by a base composed of a 500 kg inertial mass positioned over a viscous-elastic material, which isolated the system from environment vibration. At last, the capacitances of the electrodes (*C*_O_ and *C*_I_) were measured using a Fluke 8846A multimeter.

## Electronic supplementary material

Dataset 1

Dataset 2

Dataset 3

Dataset 4

Dataset 5

Dataset 6

Dataset 7

Dataset 8

Dataset 9

Dataset 10

Dataset 11
